# Fellow-Workers and the Roll of Honour

**Published:** 1915-07-03

**Authors:** 


### ROUND THE WORLD.

[The Editor invites Early Information and Contributions Marked "Fellow-Workers."

### Fellow-Workers and the Roll of Honour.

THE head of the department for anti-rabies inoculation at the Pasteur Institute, Paris, Dr. Auguste Chaillou, has fallen in recent fighting. He held the rank of médecin-major de territoriale.

HOW heavily conditions engendered by war may press upon the medical services is clearly shown by a correspondent of the *Times* (June 22, 1915), in reference to Serbia. From this communication it appears that ninety-three Serbian doctors have lost their lives out of 387 mobilised at the outset, and it is interesting to note that of these only one died on the field of battle, whilst eighty-two succumbed to typhus fever. There is no need for wonder that Serbia should have earnestly appealed for help from foreign medical men considering that she has lost about a quarter of her own medical *personnel.* Further, it appears from the same source that of the doctors who hastened to help the stricken Serbians thirty-five have died, typhus and enteric having attacked the visitors unsparingly.

IT will be remembered that Mr. James Berry, F.R.C.S., surgeon to the Royal Free Hospital, has been working hard in Serbia, whither he took a hospital mission some months ago, as was noted in THE HOSPITAL at the time, and he has had opportunities of collecting statistics which the *Times* correspondent referred to now quotes as evidence of the terrible mortality amongst doctors serving with the Serbian Army.

MAJOR JAMES CRAIK TAYLOR, R.A.M.C. (T.F.), whose name appeared in a recent casualty list amongst those who have died of wounds, was a Glasgow practitioner who for many years had taken a keen interest in the Territorial medical service. Graduating at Glasgow in medicine and surgery in 1896, he subsequently served through the South African War, being present at several close engagements; he received the Queen's medal with two clasps, and was given the rank of an honorary lieutenant in the Army. Later, settling at Newlands, Glasgow, he was for a time physician to the Stewarton Hospital. In spite of his numerous interests Major Taylor found time to publish some practical "Diet Charts for the Use of Physicians."

AN old Marlburian, Second-Lieutenant Anthony Clifford Clifford, killed in action near Ypres, had won the affection and respect of a remarkably extensive circle of those whom he had met at Guy's Hospital and Emmanuel College, Cambridge. From time to time a young man stands out amongst his contemporaries as a type of all that is manly, good, and true; Clifford was just such an one. Qualifying in 1913, he became an M.B., B.C. Cantab. the same year, but even before he had taken a diploma he had obtained a commission in the Reserve of Officers. At Guy's Mr. Clifford held all the house appointments, and when the Army was mobilised last year he joined the 3rd Cavalry Reserve. At the time of his death he was directing a machine-gun section with the 3rd Dragoon Guards.

AMONGST eminent doctors who have lately lost sons on the battlefield, sympathy goes out to Lieutenant-Colonel William Thorburn, R.A.M.C. (T.F.), whose youngest son, Lieutenant E. F. Thorburn, has been killed in the Dardanelles campaign. Lieutenant-Colonel Thorburn occupies a distinguished position amongst British surgeons; an M.D. Lond., F.R.C.S. Eng., he won three gold medals at London University examinations in his student days, and is now senior surgeon to the Manchester Royal Infirmary, and Professor of Clinical Surgery at Manchester University. Since mobilisation he has served on the staff of the 2nd General Military Hospital.

ANOTHER well-known North Country surgeon who has sustained a similar loss is Dr. George Haliburton Hume, M.D., F.R.C.S. Edin., consulting surgeon to the Royal Infirmary at Newcastle-on-Tyne. His son, Second-Lieutenant G. M. Hume, attached to the Royal Engineers (Northumbrian Division), was killed in France during the fighting last month. An old Uppingham School boy, Mr. G. M. Hume was only nineteen years of age.

AUSTRALIA has lost one of her most brilliant medical sons by the death of Captain Gordon Clunes McKay Mathison, who was wounded early in May in the Gallipoli fighting, and died at the Ras-el-Tin Hospital, in Alexandria, a week or so later. Qualifying M.B. of Melbourne University with high honours in 1905, he equally distinguished himself at the Melbourne B.S. in the following year, and then came to London, where he quickly initiated a series of brilliant researches in physiology. As a Sharpey scholar and assistant in physiology at University College Mathison closely identified himself with the general life of the institution. Being successful in obtaining a Beit Research Fellowship, he devoted himself with renewed energy to abstruse problems in physiological chemistry. However, quite soon a post was created for him at Melbourne University, to which he returned. On the outbreak of war he received a commission as captain, and was appointed to the 2nd Field Ambulance of the Australian Imperial Expeditionary Force.

THE late Mr. Edward Scott Carmichael, whose death occurred on June 12 in Edinburgh, although not on active service, nevertheless had been greatly interested in military surgery, and, indeed, held the rank of captain (R.A.M.C., T.F.) in the 2nd Scottish General Hospital. Still in the early forties, Mr. Carmichael had won for himself a fine position as an operating surgeon and specialist for children's diseases, and appeared to be destined for an exceptionally brilliant career when attacked by fatal illness. Becoming an M.B., F.R.C.S. Edin., he spent some time in Continental and American clinics before settling down to private practice. His keenness and perseverance gained him the important posts, amongst others, of assistant surgeon at the Edinburgh Hospital for Sick Children, assistant surgeon to the Royal Infirmary, and lecturer in clinical surgery at Edinburgh University.

AMONGST medical prisoners of war in Germany, as the *Lancet* reminds us, is Fleet-Surgeon Louis Greig, a well-known sportsman, who has won fame on the football field as a Scottish International player. Mr. Greig is a Glasgow University man, and graduated M.B., Ch. B. there in 1905.

